# Examining the association between peer support and English enjoyment in Chinese university students: the mediating role of regulatory emotional self-efficacy

**DOI:** 10.3389/fpsyg.2023.1278899

**Published:** 2023-12-22

**Authors:** Xiaoquan Pan, Zihan Yuan

**Affiliations:** ^1^Xingzhi College, Zhejiang Normal University, Jinhua, China; ^2^College of Foreign Languages, Zhejiang Normal University, Jinhua, China

**Keywords:** peer support, English enjoyment, regulatory emotional self-efficacy, positive psychology, English language learning

## Abstract

As positive psychology is imported into second language acquisition, we witness the increasing interest in the research of English enjoyment. Therefore, investigating the antecedents of English enjoyment is of paramount importance. Although internal and external factors have been scrutinized by researchers, scarce studies have explored the effect of peer support and regulatory emotional self-efficacy on English enjoyment. Thus, this study was designed to further clarify the association between these two constructs and English enjoyment. A questionnaire involving the three variables of peer support, English enjoyment, and regulatory emotional self-efficacy was administered to 324 non-English major undergraduate students at a comprehensive university in Eastern China. Correlation analysis and mediation effect tests indicated that peer support and regulatory emotional self-efficacy positively predicted English enjoyment, and regulatory emotional self-efficacy played a mediating role between peer support and English enjoyment. This study highlights the significance of peer support and positive emotions in second language learning and extends our understanding of enhancing students’ learning enjoyment through teachers’ positive intervention to facilitate peer support and regulatory emotional self-efficacy.

## 1 Introduction

The research of second language acquisition (SLA) has embarked on a “positive renaissance” spurred by the boom of positive psychology (MacIntyre and Gregersen, 2012; Oxford, 2015; Dewaele and Li, 2020). Since MacIntyre and Gregersen (2012) first brought positive psychology to the study of SLA, domestic and foreign language researchers have advocated a holistic view of diverse emotions in L2 learning (Jiang and Li, 2017; Dewaele and Li, 2018), which shifted the study of emotions from a singular attention on negative emotions, especially anxiety, to a more comprehensive investigation of negative and positive emotions (Dewaele et al., 2018). The study of positive emotions is crucial as they not only promote foreign language learning in terms of cognition and motivation but also lead to well-being (Saito et al., 2018). In addition, exploring the association between positive emotions and foreign language learning can contribute to language learners’ academic engagement (Khajavy, 2021). Amid the plethora of positive emotions, enjoyment, as one of the most typical emotions for language learners, is increasingly a focal point in SLA (Piniel and Albert, 2018; Jiang and Dewaele, 2019). The term foreign language enjoyment (FLE) is portrayed as “enjoyment, fun, interest, and lack of boredom” perceived in the L2 environment (Dewaele and MacIntyre, 2014, p. 242).

A growing body of research has illustrated the positive role of foreign language enjoyment. In foreign language classrooms, higher levels of enjoyment are related to higher foreign language achievement (Piniel and Albert, 2018; Li et al., 2020a), with higher levels of motivation (Wang et al., 2023) and engagement (Dewaele and Li, 2021) and with higher willingness to communicate (Khajavy et al., 2018). As confirmed by a previous survey by Dewaele and MacIntyre (2014), the enjoyment derived from the foreign language classroom motivated students to vigorously engage in classroom learning and discover the joy of unfamiliar phonological and cultural areas. In light of this, wide-ranging empirical research has been performed on the potential antecedents of foreign language enjoyment, mainly targeting a range of external (e.g., classroom environment, teacher factors, peer factors), and internal variables (e.g., age, gender, foreign language proficiency, attitudes, emotion regulation, trait emotional intelligence) (Dewaele and MacIntyre, 2014; Ahmadi-Azad et al., 2020; Li et al., 2021; Zheng and Zhou, 2022). Nonetheless, the exploration of the impact of peer support and regulatory emotional self-efficacy on foreign language enjoyment is still scant. In short, limited attention has been allocated to the role of peer support and regulatory emotional self-efficacy in enhancing foreign language enjoyment. To bridge this lacuna, the current research endeavored to unveil the influence of peer support and regulatory emotional self-efficacy on Chinese university students’ English enjoyment.

Peer support, which is subordinate to social support in the school environment (Pekrun et al., 2002), refers to students’ perceived academic concern and assistance along with emotional encouragement and affirmation from peers (Ghaith, 2002). Peer support is an important source of social support for college students and is conceptualized as an interpersonal connection established by individual college students in common activities and mutual cooperation (Lamis et al., 2016). Among a host of extant literature, the pseudo-longitudinal research in British schools from Dewaele and Dewaele (2017) examined that an array of psychological and contextual variables, embracing peer factors, can elicit fluctuations of FLE. To be specific, peer support is positively predictive of students’ foreign language enjoyment but negatively predictive of their foreign language anxiety (Khajavy et al., 2018). Adopting the three dimensions of FLE-Teacher, FLE-Peer, and FLE-Self (Jiang and Dewaele, 2019), Fang and Tang (2021), by gathering questionnaires from 140 English major undergraduates and interviewing 6 students, demonstrated that FLE-Peer was the most frequently stated source of FLE and that friendly interaction between peers ignited a passion for learning English. The aforementioned accumulating evidence converges to indicate the pivotal and distinctive position of peer support in language acquisition, especially in FLE (Leowen and Sato, 2018; Xie and Guo, 2022).

Regulatory emotional self-efficacy pertains to an individual’s belief or level of confidence in effectively managing and regulating one’s own emotions, and it mainly consists of perceptions of one’s own different emotional states, the individual’s understanding of other people’s emotions, one’s ability to manage emotions, and the corresponding different emotional responses (Caprara et al., 2008), involving two dimensions: ameliorating negative emotions and expressing positive emotions (Bandura et al., 2003). Previous research has demonstrated that regulatory emotional self-efficacy is a negative predictor of negative emotions such as depression and anxiety, generating higher subjective well-being (Pu et al., 2017; Li et al., 2020b; Li and Xie, 2021; Xiao and Huang, 2022).

However, researchers should not overly concentrate on the link between regulatory emotional self-efficacy and negative emotions as positive emotions, such as enjoyment, serve to cushion the deleterious effects of negative emotions. Language learners need to not only govern their negative emotions but also keep positive emotions, such as enjoyment, to assist the smoothness of interaction and knowledge building (Jarvenoja and Jarvela, 2009; Zhang et al., 2021; Li and Wei, 2022). For instance, Wang et al. (2021a) put forward seven positive psychological variables, covering emotional regulation and enjoyment that facilitate students’ L2 attainment and success. As noted by Jarvenoja and Jarvela (2009), students in cooperative learning utilized three kinds of emotion regulation, namely, self-regulation, co-regulation, and socially shared regulation, to address emotional challenges. Therefore, emotional regulation has a vital bearing on language learning.

Although scholars have undertaken multiple studies from external or internal viewpoints, there is a scarcity of studies tackling the influence of both internal and external variables of English enjoyment. In short, the extent to which English enjoyment may be influenced by peer support (external factor) and regulatory emotional self-efficacy (internal factor) remains underexplored. Thus, the aim of this study was to ascertain the relationship between peer support, regulatory emotional self-efficacy, and English enjoyment and whether regulatory emotional self-efficacy would mediate the association between peer support and English enjoyment.

## 2 Literature review

### 2.1 Peer support

Peer support was first applied in the area of mental health and refers to individuals who have recuperated from a mental illness and thus provide assistance to others with the same mental illness (Walker and Bryant, 2013). In recent years, researchers in the domains of pedagogy and psychology have conducted research related to peer support (Ulmanen et al., 2022; Xie and Guo, 2022), but a clear definition is lacking. In contrast, the sources and structure of social support have been more intensively analyzed (Barrera and Ainlay, 1983; Langford et al., 1997). Given that one of the essential components of social support is peer support (Wentzelkr et al., 2010), clarifying social support assists in elucidating the source and structure of peer support.

In terms of sources, social support can be obtained from family, friends, and significant others (Zimet et al., 1988). By structure, social support can be categorized into four types, namely, esteem support, informational support, social companionship, and instrument support (Cutrona and Russell, 1990). In light of the support function, social support is composed of material aid, behavioral assistance, intimate interaction, guidance, feedback, and positive social interaction (Barrera and Ainlay, 1983). In the field of education, social support can be classified into teacher support and peer support, regarding the sources of support that students receive, or academic support and personal support, contingent on the content of the support received by students (Ghaith, 2002). Grounded in the existing literature (Johnson and Johnson, 1983; Ghaith, 2002; Kim et al., 2018), peer support in this study was described in two key dimensions: (1) peer academic support, which refers to learning-related attention and assistance from peers (mainly classmates) who have similar ages, experiences, and frequent contact; (2) peer emotional support, which focuses on esteem, trust, care, and affection from peers.

In Vygotsky’s view, learning arises in a socio-cultural environment where the learner is in conversation with more competent individuals (parents, teachers, or more advanced peers) (Vygotsky, 1978). For language learners, social support, particularly from peers, provides a wide range of resources to foster learning (Gardner, 1985; MacIntyre and Gregersen, 2012). As the results of former investigations have shown, the support of peers and teachers has a favorable predictive impact on foreign language enjoyment (Khajavy et al., 2018).

### 2.2 Foreign language enjoyment

Enjoyment, as one of the most salient positive emotions, is defined as the perception of satisfaction and reward in or by the results of activities (Ainley and Hidi, 2014; Pavelescu and Petric, 2018). In the literature of positive psychology, enjoyment refers to a positive subjective feeling that an individual possesses when accomplishing some challenging or limit-pushing tasks (Csikszentmihalyi, 1990). MacIntyre and Gregersen (2012) initially introduced positive psychology to second language acquisition (SLA) research, and the past decade witnessed a robust interest in positive emotion, especially enjoyment (Dewaele and MacIntyre, 2014; Dewaele et al., 2018). In the field of language learning, foreign language enjoyment (FLE) is a “complex emotion, capturing interacting dimensions of the challenge and perceived ability that reflects the human drive for success in the face of difficult task” (Dewaele and MacIntyre, 2016, p. 216). Concerning the dimensions and measurement of FLE, Dewaele and MacIntyre (2014) were the first to exploit a 21-item Foreign Language Enjoyment Scale exploring positive emotions toward learning experiences, peers, and teachers. A subsequent study by Dewaele and MacIntyre (2016) modified the original 21-item scale to a 14-item scale, consisting of two sub-dimensions: FLE-social and FLE-private. The essential functions of foreign language enjoyment, as outlined above, hinge on positive emotions (Fredrickson, 2001) and achievement emotions (Pekrun, 2006), which stem from positive psychology. In terms of the former, positive emotions, such as enjoyment, widen the horizons of individual learners and facilitate the construction of resources, making it effective for learners to concentrate on L2 input (Mackey, 2006; MacIntyre and Gregersen, 2012). Likewise, underpinned by the three-dimensional taxonomy of the control-value theory of achievement emotions (Pekrun, 2006), enjoyment is an activation that can orient positive academic endeavors (Jin and Zhang, 2021).

Regarding the existing research on the factors affecting foreign language enjoyment, the vast majority of the current FLE research is predominantly centered on how it pertains to internal factors (e.g., age, gender, emotion, motivation, language proficiency) and external factors (e.g., teachers, peer, classroom environment) (Dewaele and Dewaele, 2017; Dewaele et al., 2018; Fang and Tang, 2021; Li et al., 2021; Wang, 2022). From a positive psychology vantage point, foreign language enjoyment helps produce a motivational effect, which can effectively enhance foreign language learning motivation (Shao et al., 2020). As such, Saito et al. (2018) also found that learners’ foreign language enjoyment significantly and positively predicted their frequency of foreign language use and academic achievement. Similarly, the empirical research of Jin and Zhang (2021), who investigated 320 high school English learners, found that foreign language enjoyment had a direct effect on English achievement and that teacher-supported and student-supported enjoyment indirectly influenced language achievement through the enjoyment of foreign language learning. In a survey of 709 Chinese primary and secondary school students, Liu and Hong (2021) found that students’ perceived enjoyment of the classroom motivated them to be more attentive, active, and willing to be engaged in English learning.

### 2.3 The mediating role of regulatory emotional self-efficacy

Regulatory emotional self-efficacy embodies a sense of self-efficacy with the degree of confidence to successfully regulate one’s emotional states. Caprara et al. (2008) subdivided self-efficacy beliefs about managing negative emotions as “beliefs regarding one’s capability to ameliorate negative emotional states once they are aroused in response to adversity or frustrating events and to avoid being overcome by emotions such as anger, irritation, despondency, and discouragement” (p. 228), and they validated the construct with reasonable reliability and validity in the samples from Italy, the United States, and Bolivia.

Available studies have confirmed a significant negative association between regulatory emotional self-efficacy and negative emotions such as depression and anxiety (Pu et al., 2017). However, the reciprocity of regulatory emotional self-efficacy on positive emotions has captured scant scholarly attention. Regulatory emotional self-efficacy allows individuals to maintain stable self-regulation and perceive more positive emotions, which benefits psychological well-being (Li et al., 2018b). It can also indirectly affect an individual’s behavior by cognition and motivation, thus playing an important moderating role (Tang et al., 2010).

Furthermore, regulatory emotional self-efficacy was found to be robust for interpersonal relationships (Caprara and Steca, 2006), and peer relationships were found to be an integral part of interpersonal relationships (Wentzel, 2017). Moreover, according to self-efficacy theory of Bandura (2001), individuals’ speculations and judgments about their ability to perform a behavior are influenced by the evaluations of people around them. In particular, the opinions of significant others can impact an individual’s self-efficacy. For instance, through an empirical study of an online collaborative English writing program, Zhang et al. (2022) explored the relationship between emotion regulation and enjoyment among language learners and exposed two forms of emotion regulation: peer regulation and group regulation. Zheng and Zhou (2022) proved that college English learners with higher emotional regulation skills have a greater likelihood of perceiving enjoyment in the learning process and that peer personal support during cooperative learning was effective in boosting foreign language enjoyment. Wang et al. (2021b) discovered that the higher the self-efficacy, the more pleasant feelings (e.g., enjoyment) and the less unpleasant feelings (e.g., shame) were perceived by non-English undergraduate majors. In conclusion, regulatory emotional self-efficacy may mediate peer support and English enjoyment.

### 2.4 Current study

Informed by the above-discussed emerging insights from recent research of peer support, regulatory emotional self-efficacy, and English enjoyment, two overarching research questions for the present study are specified below:

Question 1: What are the associations between peer support, regulatory emotional self-efficacy, and English enjoyment among non-English major undergraduate students in China?

Question 2: Will regulatory emotional self-efficacy mediate this relationship?

**Figure 1:**
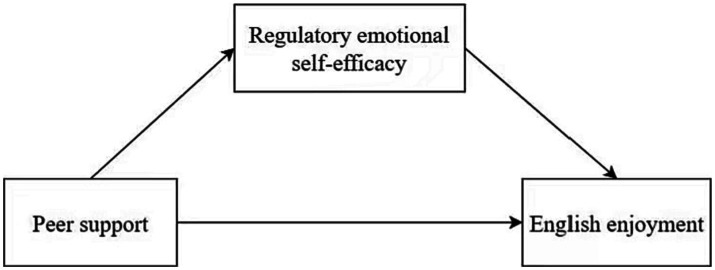
Hypothesized model of peer support, English enjoyment, and regulatory emotional self-efficacy.

Thereby, we hypothesized that there is a positive correlation between peer support and English enjoyment (Hypothesis 1); regulatory emotional self-efficacy is positively correlated with peer support and English enjoyment (Hypothesis 2); regulatory emotional self-efficacy plays a mediating role in peer support and English enjoyment (Hypothesis 3). The hypothesized model is shown in Figure 1.

## 3 Methodology

### 3.1 Participants and research procedures

A total of 345 non-English major undergraduate students studying for a college English course at a large comprehensive university in Eastern China voluntarily participated in the research. The sample was drawn from seven randomly selected classes. The 345 participants engaged in the questionnaire survey during the recess of the college English curriculum and completed it within about 10 min. The questionnaires were retrieved immediately after completion. To protect the privacy of the respondents, the questionnaire was anonymous. The sum of 324 valid questionnaires was retained, with a recovery rate of 93.91%. The age spectrum of the valid participants was 18–24 years old (*M* = 21.32, SD = 1.28), of whom 126 (38.89%) were men and 198 (61.11%) were women.

### 3.2 Instruments

#### 3.2.1 Peer support

The questionnaire on peer support, which was adapted from Johnson and Johnson (1996), served to measure perceived peer support. After deleting irrelevant items by exploratory factor analysis (EFA), the peer support scale involved a total of 12 items in two dimensions: peer academic support (e.g., “Classmates usually share their English learning methods and strategies with me”) and peer emotional support (e.g., “Classmates respect my feelings, for example, they do not laugh at me when I am not doing well in class”). Each item was assessed on a five-point Likert scale ranging from 1 (strongly disagree) to 5 (strongly agree). The higher the total score was, the better the perception of peer support was. The Cronbach’s α value of the peer support scale was 0.902. The Kaiser–Meyer–Olkin (KMO) value was 0.917, and Bartlett’s spherical test was at less than 0.001, indicating that this scale had satisfactory reliability and validity.

#### 3.2.2 English enjoyment

In this study, the questionnaire on English enjoyment consisting of 14 items was adapted from the foreign language enjoyment questionnaire used by Dewaele and MacIntyre (2014) and Li et al. (2018a) to gauge participants’ English enjoyment. A five-point Likert scale was constructed, ranging from 1 (strongly disagree) to 5 (strongly agree). Some examples from the English enjoyment questionnaire are: “I do not get bored in English class.” and “We form a tight group.” The Cronbach’s α value of the English enjoyment scale was 0.924. The KMO value was 0.917, and Bartlett’s spherical test was at less than 0.001. The scale showed good reliability and validity.

#### 3.2.3 Regulatory emotional self-efficacy

The 16-item questionnaire of regulatory emotional self-efficacy was developed by and modified from Caprara et al. (2008) and Wang et al. (2021b), and it covers two dimensions: perceived self-efficacy in expressing positive effect (e.g., “I express my pleasure when something good happens”) and perceived self-efficacy in managing negative effect (e.g., “I can keep myself away from depression when I was alone”). Responses were measured on a five-point Likert scale ranging from 1 (strongly disagree) to 5 (strongly agree). Higher scores denoted better regulatory emotional self-efficacy. In this study, Cronbach’s α value of the regulatory emotional self-efficacy scale was 0.910. The KMO value for validity was 0.919, and Bartlett’s spherical test was at less than 0.001, indicating good reliability and validity.

### 3.3 Data analysis

Firstly, descriptive statistics in SPSS 27.0 were computed to calculate the mean and standard deviation of the main variables. Afterward, Pearson correlation analysis was applied to compute the correlation coefficients to scrutinize the relevance between the main variables, which served to answer the first question. Mediation analysis was performed using Process v4.1 (Model 4) in SPSS 27.0 to investigate the mediation effect of regulatory emotional self-efficacy on peer support and English enjoyment, which was intended to address the second question.

## 4 Results

### 4.1 Common method deviation test

The Harman single-factor test was conducted in order to test for the possibility of common methodological bias as all the data were derived from the participants’ questionnaires. The data results revealed that the variance explained by the first factor was 25.46%, which was much lower than the critical value of 40% proposed by Podsakoff et al. (2003). In addition, six factors had a characteristic root greater than 1. Therefore, serious common method bias was not found in the present study.

### 4.2 Descriptive statistics and correlations

Table 1 displays the means and standard deviations of the main variables. On the basis of the classification criteria of Oxford and Burry-Stock (1995) for the Likert 5 subscale, a mean greater than or equal to 3.5 is recognized as high, a mean greater than 2.5 but less than 3.4 is considered as medium, and a mean less than or equal to 2.4 is regarded as low. Thus, in accordance with the results of the descriptive statistics, participants perceived moderate levels of peer support (*M* = 3.43, SD = 0.98), possessed high levels of regulatory emotional self-efficacy (*M* = 4.26, SD = 0.74), and experienced high levels of English enjoyment (*M* = 3.83, SD = 0.62). Pearson correlation matrices between peer support, regulatory emotional self-efficacy, and English enjoyment were also contained in Table 1.

**Table 1:** Descriptive statistics and correlation between the study variables.

Variable	Mean	SD	1	2	3
1. Peer support	3.43	0.98	(0.902)		
2. Regulatory emotional self-efficacy	4.26	0.74	0.526**	(0.910)	
3. English enjoyment	3.83	0.62	0.494**	0.543**	(0.924)

*N* = 324. **Correlation is significant at the 0.01 level (two-tailed). Reliabilities (Cronbach *α*) are shown on the diagonal in parentheses.

As delineated in Table 1, there was a positive correlation between peer support and English enjoyment (*r* = 0.494, *p* < 0.01); regulatory emotional self-efficacy was positively correlated with peer support (*r* = 0.526, p < 0.01) and English enjoyment (*r* = 0.543, *p* < 0.01), which verified Hypotheses 1 and 2. These results indicate that the higher the peer support and regulatory emotional self-efficacy, the higher the degree of English enjoyment one can experience.

### 4.3 The moderated mediation effect test

PROCESS macro program (Model 4) was conducted to further identify the mediating effect of regulatory emotional self-efficacy on the link between peer support and English enjoyment. With peer support as the independent variable, enjoyment of English as the dependent variable, and regulatory emotional self-efficacy as the mediator, a bias-corrected percentile *bootstrap* method was implemented to test for mediation effects on 5,000 replicated samples, and the results revealed that the hypothesized model’s mediation effects were remarkable (Hayes, 2017). The exact results of the mediation effect tests are shown in Tables 2, 3.

**Table 2:** The moderated mediation effect test.

Variable	Equation 1		Equation 2		Equation 3	
	*β*	*t*	*β*	*t*	*β*	*t*
Peer support	0.313	10.189***	0.395	11.084***	0.182	5.460***
Regulatory emotional self-efficacy					0.330	7.444***
*R* ^2^	0.244		0.276		0.355	
*F*	103.805***		122.847***		88.383***	

(1) Each variable in the model was standardized and brought into the regression equation. (2) Equation 1: peer support predicts English enjoyment; Equation 2: peer support predicts regulatory emotional self-efficacy; Equation 3: Peer support and regulatory emotional self-efficacy jointly predict English enjoyment. ***p < 0.001.

**Table 3:** Analysis of the mediating effect of regulatory emotional self-efficacy.

	Effect size	SE	95% CI	Ratio to total effect
Total effect	0.313	0.031	[0.012, 0.252]	
Direct effect	0.182	0.033	[0.048, 0.117]	
Indirect effect	0.131	0.022	[0.089, 0.174]	41.85%

The results of the mediation model suggest (Table 2) that peer support significantly predicts English enjoyment (*β* = 0.313, *t *= 10.189, *p* < 0.001). As both peer support and regulatory emotional self-efficacy were factored into the regression equation, both peer support (*β* = 0.182, *t* = 5.460, *p* < 0.001) and regulatory emotional self-efficacy (*β* = 0.330, *t* = 7.444, *p* < 0.001) were notable predictors of English enjoyment.

**Figure 2:**
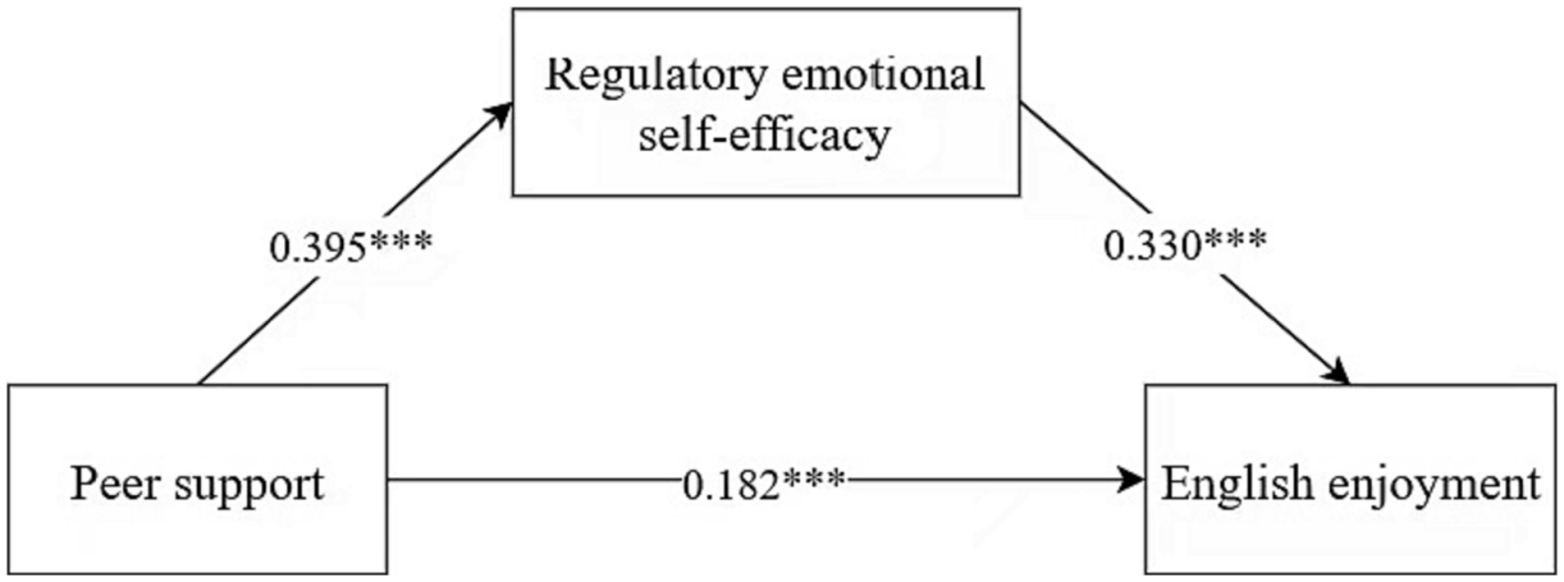
The moderated mediation effect test. ***p < 0.001.

The bootstrap test of mediating effects is shown in Table 3. The total effect and direct effect of peer support on English enjoyment and the mediating effect of regulatory emotional self-efficacy do not include 0 (from 0.012 to 0.252) in the 95% confidence interval, indicating that regulatory emotional self-efficacy has a partially mediating effect between peer support and English enjoyment; moreover, the value of the mediating effect is 0.131, and the percentage of the mediating effect is 41.85%. Figure 2 shows the mediation model, which contains the coefficient values for each relationship between the variables. Thus, regulatory emotional self-efficacy partially mediates peer support and English enjoyment, and Hypothesis 3 was confirmed.

## 5 Discussion

The preliminary aim of this study was to straighten out the correlation between Chinese EFL students’ English enjoyment with peer support and regulatory emotional self-efficacy. The Pearson correlation test uncovered, first, that a direct and profound association between peer support and English enjoyment existed and, second, that regulatory emotional self-efficacy was significantly and positively correlated with peer support and English enjoyment, which confirmed Hypotheses 1 and 2.

Regarding the evident link between peer support and English enjoyment, this discovery is consistent with that of Dewaele et al. (2018), who revealed that foreign language learners’ enjoyment is relevant to the amount of time they spend interacting with peers, and peer engagement and collaboration constitute the major extrinsic causes of foreign language enjoyment (Li et al., 2018a). It is noteworthy that this result is also aligned with the outcomes of Dewaele and MacIntyre (2014), who delved into the contributors to students’ enjoyment in foreign language classes by means of questioning in an open-ended approach, and the results showed that having supportive peers was one of the major generators of learning enjoyment. Just as the self-determination theory accentuates the effects of social and cultural contexts on people’s basic psychological needs, performance, and well-being (Legault, 2017), as a critical component of social support, peer support has been found to facilitate students’ academic and affective engagement (Furrer and Skinner, 2003; Perdue et al., 2009). On the favorable correlation between regulatory emotional self-efficacy and English enjoyment, this provides support for Zimmerman and Schunk (2011), whose research illustrated that the interplay between positive affectivity and self-regulation serves to enhance language learning achievement, happiness, and well-being. This outcome also echoes Zhang et al. (2021), who identified that three forms of emotion regulation, namely, self-regulation, co-regulation, and socially shared regulation, could bolster EFL learners’ group-level enjoyment during online collaborative language learning.

As for the subsidiary purpose, this study discovered that regulatory emotional self-efficacy acted as a partial mediator between peer support and English enjoyment, signifying that peer support not only directly impacted English enjoyment but also influenced English enjoyment via regulatory emotional self-efficacy. This suggests that peer support and regulatory emotional self-efficacy are the important antecedent variables of English enjoyment, and both have an impact on English enjoyment from the perspective of learners’ psychological environment. Meanwhile, this result also illustrates the impact role of learners’ emotions (e.g., enjoyment) as an important variable in the social cognitive domain that can have an impact on emotion self-efficacy, further confirming the influential role of emotions on self-efficacy. In addition, this also echoes the control-value theory proposed by Pekrun (2006), which postulates that self-efficacy, as a perceptual assessment of manageability, is an antecedent variable of personal emotions. Foreign language enjoyment is not only stemmed from an individual’s subjective emotional experience but also from society and involved environmental elements (Li et al., 2018a). Considered one of the external environmental factors, peer support can directly exert a positive impact on EFL learners’ positive emotions, such as English enjoyment (Ahmed et al., 2010; Chen et al., 2017; Xie and Guo, 2022), by providing academic support and emotional support (Ghaith, 2002), Favorable peer relationships are the basis for the growth of self-efficacy in emotion regulation. This result also confirms the previous findings that there is a close connection between an individual’s confidence in their own abilities and the evaluations of others (Caprara and Steca, 2006). Meanwhile, regulatory emotional self-efficacy is effective in alleviating negative emotions and restoring confidence (Li and Xie, 2021), thus perceiving more positive emotions.

## 6 Conclusion

The current study endeavored to probe the connection between peer support, regulatory emotional self-efficacy, and English enjoyment. The Pearson correlation analysis and PROCESS mediation effect test confirmed the hypothesis of this study that a remarkable positive correlation exists between peer support and English enjoyment. Regulatory emotional self-efficacy was found to correlate strongly and positively with peer support and English enjoyment. Furthermore, regulatory emotional self-efficacy partially moderated the relation between peer support and English enjoyment. Therefore, it is plausible to deduce that EFL learners who perceive more peer support and possess a high regulatory emotional self-efficacy during the learning process have a higher degree of English enjoyment. This appears to shed a light on English education. Grounded on the findings of this investigation, English educators, particularly those presently committed to teaching in an EFL context, should value the gravity and essentiality of positive emotions in second language learning. It is crucial to empower students to realize the benefits of regulatory emotional self-efficacy, to intervene in college students’ emotional maladjustment by enhancing emotional regulation self-efficacy, and to enhance their emotion regulation skills so as to experience English enjoyment. In addition, teachers’ humor and arrangement of innovative and entertaining activities (Dewaele et al., 2018; Li et al., 2021) can spark students’ interest and boost positive emotions (Fredrickson, 2001). Moreover, teachers should leverage the positive influence of peer support on college students’ emotion regulation by employing sufficient interactive and collaborative classroom activities, such as group work, role-playing, and encouraging students to interact with their peers so as to gain peer support. In conclusion, this study highlighted the significance of peer support and positive emotions in second language learning and extended our understanding on enhancing students’ learning enjoyment through teachers’ positive intervention to facilitate peer support and regulatory emotional self-efficacy.

Despite the rigorous research procedure adopted, the outputs of this investigation suffer from three major limitations. For one, the respondents of the present study were only non-English majors from one Chinese university. Therefore, caution needs to be exercised in extrapolating the conclusions to other EFL countries, and future study could expand the sample size and scope of the investigation. Next, in terms of the research methodology, a cross-sectional quantitative approach was employed, which may have yielded potential biases. It is advisable for upcoming studies to consider a longitudinal perspective for more inclusive outcomes and adopt mixed research with both quantitative and qualitative methods. Third, this study has not yet examined the variance of the main study variables on gender and age, and forthcoming research could verify this.

## Data availability statement

The original contributions presented in the study are included in the article/supplementary material, further inquiries can be directed to the corresponding author.

## Ethics statement

Ethical review and approval was not required for the study on human participants in accordance with the local legislation and institutional requirements. The participants provided their written informed consent to participate in this study.

## Author contributions

XP: Conceptualization, Methodology, Writing – original draft, Writing – review & editing. ZY: Formal analysis, Investigation, Resources, Writing – original draft.
